# Rationale, Relevance, and Limits of Stress-Induced Psychopathology in Rodents as Models for Psychiatry Research: An Introductory Overview

**DOI:** 10.3390/ijms21207455

**Published:** 2020-10-09

**Authors:** Maria Italia, Chiara Forastieri, Alessandra Longaretti, Elena Battaglioli, Francesco Rusconi

**Affiliations:** Dipartimento di Biotecnologie Mediche e Medicina Traslazionale, University of Milan, Via Fratelli Cervi 93, 20090 Segrate, Italy; maria.italia@studenti.unimi.it (M.I); chiara.forastieri@unimi.it (C.F.); alessandra.longaretti@unimi.it (A.L.)

**Keywords:** stress, animal models, stress-induced psychopathology, neuropsychiatric disorders, construct validity, face validity, predictive validity

## Abstract

Emotional and cognitive information processing represent higher-order brain functions. They require coordinated interaction of specialized brain areas via a complex spatial and temporal equilibrium among neuronal cell-autonomous, circuitry, and network mechanisms. The delicate balance can be corrupted by stressful experiences, increasing the risk of developing psychopathologies in vulnerable individuals. Neuropsychiatric disorders affect twenty percent of the western world population, but therapies are still not effective for some patients. Elusive knowledge of molecular pathomechanisms and scarcity of objective biomarkers in humans present complex challenges, while the adoption of rodent models helps to improve our understanding of disease correlate and aids the search for novel pharmacological targets. Stress administration represents a strategy to induce, trace, and modify molecular and behavioral endophenotypes of mood disorders in animals. However, a mouse or rat model will only display one or a few endophenotypes of a specific human psychopathology, which cannot be in any case recapitulated as a whole. To override this issue, shared criteria have been adopted to deconstruct neuropsychiatric disorders, i.e., depression, into specific behavioral aspects, and inherent neurobiological substrates, also recognizable in lower mammals. In this work, we provide a rationale for rodent models of stress administration. In particular, comparing each rodent model with a real-life human traumatic experience, we intend to suggest an introductive guide to better comprehend and interpret these paradigms.

## 1. Introduction

Neuropsychiatric disorders are common and disabling pathological conditions that diminish the quality of life and reduce psychosocial and executive functioning [1]. From the clinical point of view, mental disorders represent one of the major challenges of modern medicine, affecting a discrete portion of the western countries’ population, with incidences resembling those of an epidemic more than a syndromic domain [2]. In the general population, 10% to 20% suffer from these pathologies that include depressive disorders, several forms of clinical anxiety including generalized anxiety, trauma, and stressor-related disorders such as post-traumatic stress syndrome (PTSD), obsessive-compulsive and related disorders, schizophrenia, eating disorders, and addiction [1]. Neuropsychiatric disorders often involve the social sphere and environmental adaptability, displaying an aberrant general perception of the social and physical world [3]. 

Relevantly, disease classification is mainly based on symptoms analysis: as a result, it may be difficult to establish both an objective and unambiguous diagnosis. Despite many efforts undertaken to refine and standardize diagnostic procedures, univocal diagnoses may be critical since neuropsychiatric symptoms tend to cover a wide spectrum, often partially overlapping with those of other psychiatric conditions. However, symptoms-based diagnosis currently seems to represent the only option in clinical neuropsychiatry given that (i) objective and easily detectable morpho-structural brain correlates are difficult to retrieve if not denied, and their utility is limited as they are not unequivocally linked to specific behaviors; (ii) no biomarkers have yet been extensively validated to clinical purposes; (iii) incisional biopsies are not routinely performed within the central nervous system (CNS). With respect to this last point, great hope has been placed on the neuroimaging field [4], which might provide new means to correlate brain abnormal features and lesions to specific psychiatric symptomatology [5,6,7,8]. Entrained with technology evolution — in particular in the field of computed tomography (CT) and magnetic resonance (MR) — a huge body of literature has identified morphostructural determinants coupled to neuropsychiatric disorders. Often gray matter loss, leading to decreased volumes of important cognition and emotion-related brain areas including the hippocampus, dorsal anterior cingulate, prefrontal cortex, and insula, among others, represents a signature of anxiety, obsessive-compulsive disorder, depression, bipolar disorder, schizophrenia, as well as addiction. However, it is still difficult to discriminate between different forms of mental disorders by means of specific neuroimaging-retrieved structural correlates [9]. Strategies have been proposed to find a way to override these issues, including the conceptualization of brain function not only from the structural but also from the functional network architecture perspective [2]. Indeed, a specific behavior involves a set of brain systems (a brain network) that are represented by functionally independent but interconnected regions, often with a specific cytoarchitectural pattern [10,11]. In other words, cognitive, emotional, motivational, and behavioral functions rely on tightly spatially and temporally coordinated interactions among large neuronal clusters belonging to highly specialized brain areas. Characterizing human brain networks through functional multimodal imaging approaches, involving functional MR imaging (fMRI), positron emission tomography (PET), and diffusion tensor imaging (DTI), turned out to be a powerful way to compare brain network functionality of a large number of healthy subjects to that of neuropsychiatric patients, eventually revealing that defects in functional integration, as well as aberrant connectivity underlie neuropsychiatric disorders [12].

Therapeutic management of neuropsychiatric disorders is challenging. Symptoms relapse and recurrence often follow drug withdrawal; furthermore, a significant subpopulation of patients do not, or only partially respond to treatments [13]. As a matter of fact, despite the great effort devoted to neuro-psychiatric pharmacological research, no major improvements have been achieved to counteract the onset and progression of mental illness: today, like 50 years ago, most therapeutic strategies simply aim at increasing mono-amine levels in the CNS [13].

These issues mainly result from a lack of complete and comprehensive molecular, cellular, and circuitry understanding of psychiatric disorders pathophysiology, in which both genetics [14,15] — as suggested by their hereditary component [16] — and the environment plays an important role [17]. According to the Diathesis-Stress Model, diathesis or genetics are not sufficient per se to cause the majority of neuropsychiatric disorders, but it is the combination of a pre-existing genetic vulnerability with a stressor — the combination of nature and nurture — that leads to their development [18]. However, this model must be applied with a certain caution because not every patient suffering from psychopathology necessarily experienced particular stressful situations at a certain moment of his/her life. Although prenatal stress could explain some clinical cases without a clear stressful history, it cannot account for all of them. Indeed, besides stress, many other clinically-relevant factors should be coupled to genetic predisposition within the pathogenesis of mental illness. For instance, maternal immune activation in vulnerable windows of fetus development [19], maternal emotional problems during pregnancy [20], gestation issues, maternal smoking during pregnancy [21], perinatal complications, fever, and illness during the first year of life can foster [22], together with still elusive categories including toxic factors and comorbid pathologies [23,24], the onset of neuropsychiatric disorders. 

All this said, given the complex framework in which neuropsychiatric disorders are involved, any advancement in the understanding of the molecular and cellular mechanisms behind them could contribute to the improvement of their diagnosis and treatment. 

The first attempt to correlate dysfunctions of specific brain areas with psychiatric conditions came from last century studies, when researchers and clinicians observed alterations in the emotional or cognitive sphere of patients who showed brain tumors or traumatic injuries, either ischemic or mechanical, in specific brain areas. We must cite the case of Phineas Gage who, upon injury to his frontal lobe, completely changed his personality in such a profound way that his friends saw him as “no longer Gage”, thus sparking an intriguing debate regarding the function of the brain area where he had reported the lesion. However, even if these lesion-behavioral phenotype correlations provided a thought-provoking starting point for neuropsychiatric research, systematic studies that aim at identifying the pathophysiology of neuropsychiatric disorders do require animal models. 

This review discusses the rationale of using environmental stress as a strategy to recapitulate some endophenotypes of human stress-related psychiatric disorders in rodents. As an essential premise, we must highlight that no animal model of psychopathology obtained by environmental stress administration can closely reproduce all the features of a human disorder: in the best-case scenario, these models can display some endophenotypes of human psychopathologies, showing a low disease-to-model correspondence.

Nonetheless, rodent models acquired renewed relevance in investigating the molecular and pathophysiological mechanisms underlying mental disorders, thanks to the introductions of Research Domain Criteria (RDoC), https://www.nimh.nih.gov/research/research-funded-by-nimh/rdoc/about-rdoc.shtml, a new framework to approach the study of psychopathologies. In this regard, RDoC, based on domain analyses of easily identifiable specific symptoms, has been proposed to couple behavioral signs present in both humans and rodents, with inherent molecular substrates. This approach overrides the requirement of a good disease-to-model correspondence, enhancing the significance of data obtained from animal models but also their translational potential. 

From the clinical point of view, neuropsychiatric disorders are classified following the Diagnostic and Statistical Manual of Mental Disorders (DSM-5) guidelines. While the DSM-5 represents a standardized diagnostic tool, taxonomically holding disease-specific criteria that are used to provide diagnosis, treatment, and therapy [1], RDoC classification identifies six major domains of human functioning. These domains (negative valence, positive valence, cognitive systems, systems for social processes, arousal or regulatory systems, and sensorimotor systems) contain several behavioral elements, or constructs, that comprise different aspects of the overall range of functions, from normal to abnormal. These constructs are associated with specific, measurable neurobiological elements, also known as neuropsychiatric endophenotypes. Specifically, each construct can be analyzed at different levels, which include molecules, cells, circuits, and physiological, behavioral, and self-reported assessments [25]. In conclusion, RDoC are not as diagnosis-oriented as DSM-5, being more focused on facilitating translation from basic neuroscience research findings into pathogenic mechanisms and the development of new therapeutic approaches [26]. 

## 2. From Stress to Neuropsychiatric Disorders

Environmental stress is the result of the subjective perception of a potential or manifested threat. From the biological point of view, environmental stress triggers the activation of different transduction cascades that finally modify an individual’s psychophysical equilibrium, thus impacting behavior and cognitive performances. It is possible to identify two temporally distinct sets of stress responses. The initial perception of a stress triggers reactions whose aim is to prepare the body to respond to the threat (both invigorating physical strength and cognitive acuity): this is called primary or “fight-or-flight” response. Such a primary response is followed by homeostatic feedback that is more related to avoiding excessive reaction and to promote stress response termination aiming at restoring an equilibrium typical of resting conditions [27].

**Figure 1:**
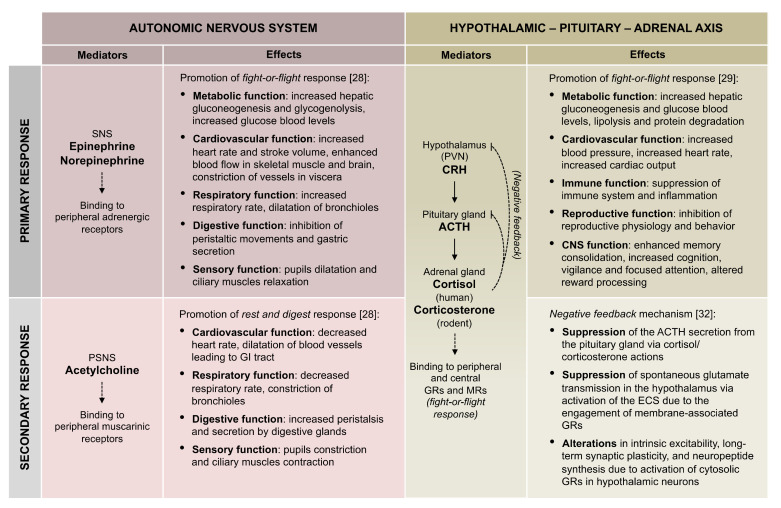
Primary and secondary autonomic nervous system (ANS) and the hypothalamus-pituitary-adrenal (HPA) axis response to stress. In the table, the molecular mediators and the main effects of primary and secondary responses to stress of ANS and HPA axis are reported. In both systems, the primary response promotes fight-or-flight reaction to properly face the perceived stress. Conversely, different secondary response mechanisms are adopted: ANS actively promotes rest and digest response through the activation of PSNS, whereas HPA axis secondary response consists of negative feedback mechanisms guaranteeing the recovery of resting conditions-inherent axis responsivity. SNS (Sympathetic nervous system); PSNS (Parasympathetic nervous system); PVN (Paraventricular nucleus); CRH (Corticotropin releasing hormone); ACTH (Adrenocorticotropic hormone); GRs (Glucocorticoid receptors); MRs (Mineralocorticoid receptors); CNS (Central nervous system); ECS (Endocannabinoid system).

The principal neuroendocrine processes involved in stress response are the autonomic nervous system (ANS) and the hypothalamus-pituitary-adrenal (HPA) axis [28,29]. Importantly, in mammals, these systems and their functions are highly conserved. The ANS and HPA axis are involved in triggering both primary and secondary responses. On the one hand, their activation supports all the psychophysical changes that prepare the body to fight-or-flight [30,31]. On the other hand and coherently with the general principle exposed above, these systems are also provided with intrinsic mechanisms of negative regulation that guarantee, in healthy individuals, their limited and transient activation (secondary response) [32]. Notably, in addition to feedback components of the ANS and HPA axis, played within a multisystemic and complex interaction among the central and peripheral nervous systems and multiple endocrine glands (Figure 1), at the cellular level, two other mechanisms display similar homeostatic features, including the endocannabinoid system (ECS) [33,34,35,36,37] and a set of epigenetic responses to stress aimed at restraining neuroplasticity-related gene transcription [38,39,40,41,42]. These two systems belong to a complex group of synapse-to-nucleus cross-talk mechanisms aimed at controlling glutamatergic signaling and its transduction, in the frame of diminishing excessive stress-responses at the neuronal level [34,39,43,44]. Consistently, within a more comprehensive vision, the stress-response process is better defined by the concept of allostasis [27,45,46]. Allostasis means “remaining stable by being variable”, and it refers to the process of “achieving stability, or homeostasis, through physiological or behavioral changes*”* [47]. According to this definition, the outcome of the stress transduction is the achievement of an equilibrium that is generally different from the original one representing a new form of stability. The reason why allostatic mechanisms exist is to guarantee adaptation and habituation to environmental changes: Processes that require metabolic energy expenditure to undertake neurochemical and behavioral changes aimed at adapting to a new external condition, ultimately to survive [46]. This happens through the release of specific mediators that act on specific neuronal populations localized in different cerebral areas or peripheral nervous system [48]. A relevant consequence of allostasis in healthy stress response is that if a second but similar stress occurs, the body sensitivity and its psychobiological responses are primed to react differently with an adaptive mean related to habituation or desensitization. However, stress responses are not always adaptive [49]. Depending on the stress nature, intensity, duration, but most of all, individual susceptibility under the form of genetic (innate) or epigenetic (acquired) predisposition, it can happen that an individual undertakes maladaptive trajectories, including neuropsychiatric drifts [50,51]. In other words, persistent (i.e., chronic) or excessive stress can corrupt allostatic mechanisms at the molecular level, leading to overload [38,52]. The overload can induce toxic neurobiological modifications in stress-transduction mechanisms impeding, under certain circumstances, adaptive stress-responses [53]. Crucially, the new maladaptive equilibrium can persist for a long time, even after the initial toxic stress has ended, thus heavily impacting individual psychological health and eventually leading to neuropsychiatric disorders. With respect to this issue, it has been shown that prolonged and intensive stress can precipitate pre-existing but not yet evident psychiatric tendencies, being also able to promote ex novo psycho-pathological profiles [25,53,54]. By virtue of these observations, stress administration is considered one of the best strategies to generate animal models that recapitulate specific phenotypes and endophenotypes typical of human neuropsychiatric conditions included within the RDoC [25,26]. As stated above, the employment of mammals (especially rodents) in neuropsychiatric research is meaningful ([55,56,57,58], also because environmental stress produces responses that are evolutionarily conserved in the mammalian class, in particular for what concerns neuroendocrine and autonomic systems, including the ANS and HPA axis [31].

In the following paragraphs, we will describe animal models of stress and will discuss paradigm rationale and validity in representing different aspects of mental disorders. We will also provide a perspective on how stress-paradigms currently used in neuropsychiatric research can represent a valuable proxy to study these diseases and their still elusive underlying pathomechanisms.

## 3. Validity Assessment of Rodent Models of Environmental Stress

In the field of behavioral neuroscience, animal models are used to describe and study causes, mechanisms, and symptomatic traits of human pathologies. However, these models can only partially resemble the complexity of human behaviors. In this regard, a fundamental and recent implementation of new criteria to circumvent this issue entails de-structuring human pathologies into discrete behavioral and circuital endophenotype. In other words, nowadays, the new aim of animal models is no more to recapitulate a diseas*e,* rather some of its endophenotypes [26]. 

In further detail, the process of animal model reliability evaluation (where animal model refers to animals administered with different stress paradigms) can be performed looking at three main parameters: construct validity, face validity, and predictive validity [59]. Construct validity indicates the degree to which the candidate model recapitulates the etiopathological causes of the human disorder it intends to simulate, including behavioral and pathophysiological components [60]; face validity indicates the compliance of the rodent model to disease symptomatology in humans; the pathological phenotype is evaluated in terms of molecular, neurochemical, circuital, anatomical, and behavioral determinants [60]; predictive validity measures pathological phenotypes responsiveness to pharmacological (mainly monoaminergic antidepressant and anxiolytics) therapies that have proved to be effective also in humans. If a model shows a high predictive validity, it could be used in comparative studies that aim at evaluating the efficacy of new therapeutic approaches [61] to be then tested, after preclinical validation, in clinical settings.

It must be observed that the same animal model may show diverse scores among the three above mentioned criteria, and generally, no single model shows high validity in all three parameters [59]. Despite their scientific value, animal models of stress cannot be considered models of neuropsychiatric disorders: They simply represent an aid, though the best currently available, for the study of these pathological conditions. This is why it is better not to refer to as animal models of depression or schizophrenia, but rather to an animal model for the study and comprehension of depressive and schizophrenic traits.

## 4. Animal Models of Stress, Feasibility, and Reliability as a Proxy for Mental Disorders

Two essential criteria must be matched in order to generate an animal model in general. The first criterion is that the animal should be characterized by ease of experimental use and affordable maintenance. The second criterion is that the animal must show a neurological development that is complex enough to adequately respond to paradigms than mimic human traumatic experiences. Concerning this last point, bioethical issues and very complex management related to the employment of species evolutionary closer to humans must be considered. Mammalian species that better fulfill these criteria are rats and mice. For what concerns experimental feasibility, they are characterized by small dimensions and short generation time (10 weeks). Once adults (2–3 months of life), they generate numerous litters (an average of six pups per litter, 5–10 litters per year). Nonetheless, they show adequate neurological complexity to recapitulate endophenotypes observable in humans and neurochemical stress reactions. In addition, the availability of inbred strains (i.e., individuals which are virtually genetically identical to each other) allows the isolation of the environmental component and to obtain results that are not influenced by inter-individual variability. Notably, mouse genetic manipulation (i.e., generation of knock-out (KO), knock-in (KI), and transgenic animals) allows investigating the contribution of specific allelic variants to stress susceptibility and resiliency. 

In the generation of rodent paradigms of stress-induced psychopathology, it has been successful in mimicking events that happen in nature under certain conditions, which led to their wide employment in neuropsychiatric research [62]. In accordance with these general criteria, as mammals, we mainly experience two typologies of adversive paradigms, i.e., physical and psychological stress. Physical stress model conditions of total loss of control, triggered by traumatic events such as accidents or natural catastrophes. These conditions, which can precipitate PTSD in humans, tend to lead to similar severe long-term behavioral alterations also in rodents [62]. Psychological stress ethologically challenges crucial hierarchical parameters of social interaction such as social stratification, dominance, and submission. In these cases, psychological stress falls under the category of psychosocial stress [63]. However, psychological stress can also be related to wildlife survival, where predation represents one of the more dangerous sources of life-threatening paradigms [64]. Consistently, predator perception induces heightened vigilance and fear, triggering neuroendocrine and behavioral responses and prototypical psychological stress responses [65].

Interestingly, the developmental stage of stress perception, as well as sex, strongly influences neuroendocrine, neuroplastic, and behavioral modifications. Thus, the responses and long-term consequences of the described typologies of stress can be further characterized depending on their administration stage within early life, adolescence, or adult ages [66,67]. For what concerns early-life stress, it has become clear how increased neuroplasticity and circuitry excitability, together with enhanced learning ability, correlate with increased stress vulnerability in younger mammals [66]. However, in more recent years, also adolescence has been scored as a heightened plasticity and malleability window [68]. In this regard, as in neonatal life, also within adolescence, increased circuitry plasticity not only boosts opportunities for individual growth and cognitive development but also holds relevant vulnerability aspects in particular stress-related. Recent data indeed show enhanced detrimental effects of social isolation as well as reduced fear extinction that are consistent with adolescence as a sensitive period for the development of mental illness [68]. Another very important aspect that has to be considered when investigating the effects of stress on brain circuitry and behavior is the gender effect [67]. Although all developmental and gender-related components play a crucial role in defining long-term effects of stress administration, a detailed discussion of these aspects falls beyond the scope of this review.

For both physical and psychological stress typologies, the more a stress paradigm is unpredictable and unavoidable, the more it will be effective in reproducing the expected behavioral traits [69].

In the next paragraphs, we will examine different rodent paradigms of stress that are used in current research to investigate the molecular basis of neuropsychiatric disorders and eventually to validate or discover new therapeutic strategies for their treatment. For each paradigm of stress, we will discuss the human traumatic experience that it intends to mimic, justifying, in other words, its construct validity. Such a systematic comparison of each rodent model presented with a real-life human traumatic experience aims at providing the reader who approaches the topic of rodent models in neuropsychiatry research with an intuitive key to correctly interpret the underlying rationale. In the frame of evolutionary conserved molecular, behavioral, and pathological responses that a stress paradigm elicits in rodents, we will discuss the extent to which these responses recapitulate those experienced by humans. This feature is named face validity and defines how a rodent model of stress reflects a human clinical condition. Face validity is highly relevant to choose the best typology of stress that models a specific endophenotype. Notably, it is always important to remember that a rodent paradigm of stress can, at its best, model only some aspects of a human psychiatric stress-related drift. However, we need to capitalize on the ability of such a paradigm to provide a proxy to study mentioned molecular, circuital, and behavioral endophenotypes, recapitulated on a case-by-case basis by each stress trial.

**Figure 2:**
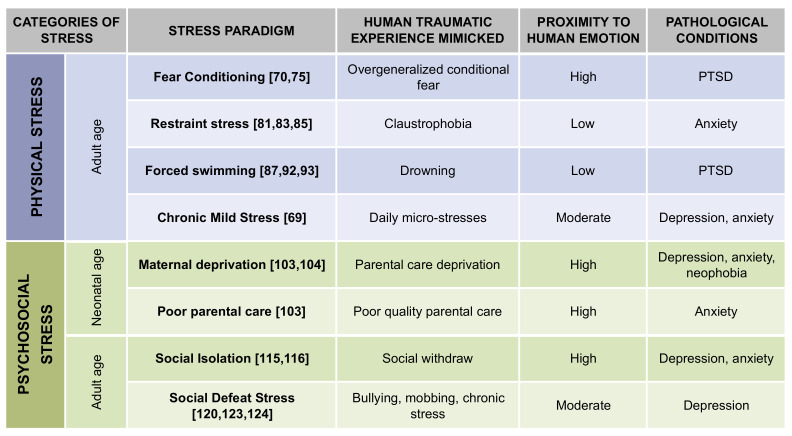
Summary of mentioned stress paradigms. The table shows the main features of mentioned stress paradigms in terms of stress category; human traumatic experience mimicked, proximity to human emotion, and pathological conditions. PTSD (Post-traumatic stress disorder).

We will also provide some experimental details and provide some hints on the preclinical relevance of rodent paradigms of stress-induced psychopathology (Figure 2).

## 5. Physical Stress

### 5.1. Fear Conditioning

Fear is defined as an unpleasant emotion caused by a perceptual sensory experience to be under threat of danger or harm. Conditional fear is a particular learning typology that associates contextual details to the fear itself, aimed at helping to recognize potential threats. Generalizing, the conditional fear represents a further behavioral response to threat regulated by a fundamental neurobiological process that aims at increasing individual survival chances. According to this mechanism, an individual identifies a certain environmental context as potentially dangerous whenever it recalls (albeit being only reminiscent, not exactly the original contextual cue) a previous traumatic event, generating avoidance of the predicted dangerous situation [70]. Overgeneralized conditional fear is the result of an aberrant, excessive association between a fear that derives from a real and well-defined traumatic event (unconditioned) and a contingent stimulus that is emotionally neutral (conditioned). In this way, the sole perception of the conditioned stimulus, in the absence of a real threat, will trigger a fear response. Overgeneralized conditional fear is a core symptom of traumatic psychopathologies [70]. For example, one of the DSM-5 criteria for PTSD diagnosis is the presence of severe emotional distress or physical reactions to events that resemble the original trauma. Various categories of traumatic stress (natural catastrophes, violent accidents, but also psychosocial stressors) can foster PTSD onset. A common PTSD crisis is triggered by the presence of a conditioned stimulus (such as noises, cries, violent and rapid movements) that, by virtue of conditional learning, evokes in the patient the memory of a previous traumatic event. As a result, the affected individual suffers from devastating anxious arousal. From an external point of view, the patient’s reaction is excessive and out of context since the unconditioned stimulus is indeed absent.

Overgeneralized Conditional Fear, and the associated pathological conditions, such as PTSD, can be at least partially modeled in rodents through the administration of specific paradigms of stress. For example, the foot shock paradigm reproduces some of the core symptoms of PTSD, such as hyperarousal, avoidance, and anxiety behaviors, together with sleep disturbances [71]. More in detail, this paradigm is based on the simultaneous administration of a traumatic stimulus such as a weak electric shock — the unconditioned stimulus — and a neutral one, such as a colored light or a tone — the conditioned stimulus. This phase is defined as associative training. Importantly, the environmental context in which the animal experiences the traumatic and neutral stimuli is itself included within the generalization process and is indicated as associative context. The unconditioned stimulus triggers in the animal a peculiar, measurable (in seconds) stereotypical behavior that reflects its fear: the freezing behavior displayed by mice and rats. Associative training can be performed once or repeated, and typically consists of reiterated pairings of neutral stimulus with an aversive one [72]. Training is successfully completed when the neutral stimulus in terms of the colored light, the tone, or even the associative context can alone trigger the freezing behavior [73]. It is worth underlining that the foot shock paradigm performed in rodents, like all the other stress-based rodent models of neuropsychiatric disorders, only partially reproduces pathological traits of PTSD in humans. It is as well worth saying that this rodent stress paradigm displays a limited construct validity. Indeed, because of the nature of the stressor (mild electric shocks), this model does not perfectly recapitulate usual PTSD etiological determinants in humans. However, because the fear conditioning paradigm does its best to recapitulate not only human PTSD symptomatology but also its principal biochemical and neuropathological features, it is considered to match high face validity requirements [74]. Another major advantage of this paradigm is that it represents an extraordinary model for pharmacological research (potential predictive validity) [74]. For example, it has been demonstrated that freezing time diminishes in response to antidepressant drugs that enhance fear extinction [75]. It is important to note that fear conditioning is often used with the aim to measure associative memory in mice [76,77,78]. To this extent, the unconditioned stimulus, i.e., foot shock, is administered to establish how strong its memory will be associated with that of a conditioned stimulus. In this frame, researchers are not necessarily interested in investigating neurobiological mechanisms of stress response impacting mental health; rather, they take advantage of the considerable strength of foot shock as a stimulus, to evaluate a “cognitive” parameter in genetically-modified of pharmacologically-treated mice in order to assess whether genotype, or treatment affect associative memory formation. Also in this case, freezing time is taken as a direct memory read out. The more a mouse will freeze once exposed to the conditioned stimulus, the more it has consolidated an associative memory.

### 5.2. Restraint Stress

Claustrophobia, literally phobia of closed-in spaces, represents a human physiological and innate form of fear. It is an adaptive emotion that increases survival chances since it promotes the search for safe spaces in which free movements are fully allowed, and the consequent refusal of narrow, hideout, potentially anoxic places. One possible example of a dramatic human situation in which restraint can be experienced is represented by earthquakes. Notably, survivors who were trapped in rubble during earthquakes are at risk of developing acute and post-traumatic stress disorders and depression [79,80]

Restraint stress, in other words impeding animal movements, can be adopted to recapitulate in rodents claustrophobia-reminiscent stress [81]. Interestingly also in rodents, body restraining entails an increased risk of developing long-term behavioral effects [82]. In this regard, it is worth noting that the comparison of human claustrophobia with the fear experienced by restrained rodents may be improper to some extent. Indeed, rodents feel more comfortable than humans in narrow spaces where they clearly experience a sense of protection. However, we suggest that the construct validity of immobilization — as a means to induce psychological stress in rodents useful to study molecular underpinnings of common endophenotypes among the species — may be at least in part supported by shared circuital substrates underlying mammalian fear.

Restraining animals in narrow spaces is another form of stress that triggers the activation of neuroendocrine systems such as HPA and ANS that allow the fight-or-flight response [83]. In particular, restraint stress not only determines an increase in serum corticosterone concentration, but it also mediates downregulation of the prefrontal cortex (PFC) glucocorticoid receptors GRs [82]. This latter result is of great relevance considering that GR downregulation has been involved in the pathophysiology of depression [84]; consequently, it may support chronic stress-induced anxiety- and depression-like behaviors that follow restraint stress administration. This paradigm of stress consists of maintaining the animal in a narrow transparent cylinder for a period spanning from 30 min to 24 h and can be administered in a single or in multiple sessions in its chronic form. In order to observe long-term behavioral issues in rodents, reminiscent of those observed in earthquake survivors, longer administrations are needed [85]. For instance, after a 24 h restraint stress, mice showed depressive-like behaviors, including a decreased preference for sweetened water (anhedonia) and increased immobility time in the forced swim test, as well as increased surrender-like behavior in terms of floating behavior. Interestingly, as molecular and cellular underpinnings, long restraint stress decreased glucose uptake in the brain and reduced hippocampal neurogenesis together with molecular modifications at the transcriptional level starting from two days after the stress, and lasting for more than one month [85]. Relevantly, immobilization paradigms have the great advantage of being relatively simple and standardizable, although construct validity is limited as it only recapitulates the immobilization phase of a natural catastrophe such as an earthquake, consequently featuring high habituation rates [86].

### 5.3. Forced Swimming

Drowning represents a relevant innate human fear. In 2015 the World Health Organization estimated 360,000 people died from drowning, representing the third leading cause of unintentional injury death worldwide. The stress paradigm of forced swimming could model, to some extent, the threat of being stuck in a small space, enhanced by the dangerousness of water, which represents a potential risk for all terrestrial mammals. In the paradigm of forced swimming, a mouse or a rat is placed into a cylindrical container filled with water at a controlled temperature. Despite rodents having an innate ability to swim and do not really risk drowning, as they cannot exit from the water, this paradigm simulates a fear that, although not univocally, we suggest might be reminiscent of that of drowning in humans. This paradigm can be administered in a single or in multiple sessions, thus recapitulating both acute and chronic stress [87]. Notably, forced swimming has an important effect on the neuroendocrine system [88]. For example, forced swimming mediates a robust corticosterone response [89] but also other neurochemical and immune changes [90]. For these reasons, its chronic administration via multiple sessions leads to anxious and depressive stereotypical behaviour [91]. The readout of these behaviors is the relative time the rodent spends floating compared to the time spent swimming hence trying to escape the stressful condition. After chronic administrations, animals progressively give up the attempt to exit from the container engaging a peculiar surrender-like behavior (anticipated and increased time floating) [92]. We would like to underline how within this specific paradigm, the same procedure is used to induce a depressive-like behavior and to measure this behavior by means of the same parameter, i.e., floating time. This is the reason why forced swim stress represents a gold standard to assess the antidepressant potential of new drugs [93], showing high predictive validity. 

## 6. Chronic Mild Stress

Until now, we have described homotypic chronic stress typologies (i.e., paradigms of stress in which the same typology of stress is administered many times). These reiterated paradigms are based on the evidence that a single stressful event (or few sessions of the same stress), as long as its severity does not overpass a traumatic threshold, does not trigger long-term consequences at the behavioral level in animal models. It is indeed the exhausting nature of multiple stressful sessions that more often leads to long-term pathological alterations.

Rodent homotypic stress paradigms well satisfy the above-described parameters used to assess the face and predictive validity of animal models. However, they do not always perfectly reflect what generally happens in human lives, showing limitations in construct validity. In fact, an individual is more likely to face many stresses of different typology rather than the same stress repeated many times, and it is the concurrence of these mild unpleasant events that more probably result in cumulatively toxic stress likely leading to pathological consequences. 

Chronic Mild Stress (CMS) paradigms aim at modeling more realistic forms of stress that results from the sum of different mild negative events. Thus, to certain extents, the strategy of increasing the nature of stress variability improves construct validity of the animal model. In CMS, unpleasant, albeit mild stresses (each typology highly efficient at triggering emotional fear, and also chronically administrable per se), are randomly reiterated for some weeks in order to enhance their pathogenic potential. The unavoidable and unpredictable nature of CMS makes this kind of stress particularly effective in triggering a depressive-like phenotype. This includes in rodents, increased readouts of anhedonia evaluated with the sucrose preference test, decreased sociability, and increased surrender-like behavior, and learned helplessness in the forced swim and tail suspension tests [55]. Examples of mild stress that can be chosen to build up a CMS protocol are the daily change of cage spatial position, the exposure to unpleasant noises, the sudden modification of light-dark cycles, the temporary removal of litter, and food administration with a random timing and not ad libitum [69]. Several studies indicate that CMS strongly affects HPA axis activity. For example, it has been found that CMS mediates an increase in hypothalamic corticotropin-releasing hormone (CRH) mRNA [94], also impacting CRH neuron density [95] and, consistently, serum corticosterone levels [96]. Coherently with the important activation of the HPA axis that follows CMS, this paradigm of stress often results in anxiety phenotypes with depressive traits [59]. CMS is administered to investigate the molecular bases of neuropsychiatric disorders. In particular mood disorders (high face validity), but also to test new antidepressant drugs (potential predictive validity). For its significant construct validity, CMS paradigms have become, in recent years, first-choice paradigms to study neuropsychiatric disorders. In this respect, CMS has been used in more than 1300 published studies: a considerable result if we consider that it was introduced only 30 years ago [69].

## 7. Psychological Stress

As we said, environmental stress stands at the interface between a relevant risk factor and a structured etiologic determinant in neuropsychiatric disorders. Although physical stress is heavily involved in precipitating disease states, in particular PTSD, luckily, not everybody will experience earthquakes, plane crashes, war, or car accidents. Vice versa, psychological stress predominates in our societies, representing a necessary and unavoidable experience that we all have to learn to cope with. Psychological stress pervades our life from infancy to old age. Parental care may be inadequate in the first years of life, then within school ages and adolescence, different forms of bullying can be experienced. Adulthood is similarly not spared, being mobbed at the workplace, and different forms of betrayal, classical stressful paradigms with potentially severe implications in mental health stability. Loss of beloved ones and loneliness (albeit possible within any age) sadly afflict aging.

### 7.1. Maternal Deprivation

John Bowlby (1907–1990) is a British psychiatrist famous for his seminal work that brought to the formulation of the Attachment theory. According to this theory, infants have an innate necessity to establish emotional ties with other individuals. This represents a mechanism of adaptation whose final goal is again to increase survival chances [97]. In particular, the relationship with the mother represents a natural haven from which to leave for world exploration and, even more importantly, the prototypical social relationship. For these reasons, in the case of maternal care deprivation, the child can incur both serious behavioral alterations of the social sphere and cognitive problems [98,99]. Commissioned by the World Health Organization to study the emotional development of children orphaned during the World War II, Bowlby thoroughly contributed to the field publishing in 1951 the pioneeristic and famous report “Maternal Care and Mental Health” [98] where he coined for the first time the term “Maternal Deprivation”[100]. Interestingly, he observed that even well-nourished orphans displayed an increased tendency to depression and other emotional and behavioral problems when compared to children that did not experience maternal deprivation. Many theories of developmental psychology — including Bowlby’s Attachment theory — have been conceived in the years immediately after last century world wars. This correlation does not appear to be incidental: these two tragic events, with their massive numbers of deaths, created large numbers of orphans whose emotional traumas frequently ended up in psychopathological conditions later in life [101]. All these young orphans represented indeed relevant cohorts to conduct important research on clinical aspects of parental deprivation. 

Rodent models of maternal separation proved that Attachment theory also applied to lower mammals. Indeed, similarly to humans, maternal deprivation can also exert in mice and rats negative long-term behavioral abnormalities, including cognitive deficits, increased susceptibility to drug addiction, and depressive and anxious phenotypes [102]. The fact that maternal deprivation has an impact on rodent behavior supports the use of this paradigm to deepen our knowledge of the molecular and circuitry that correlates underlying behavioral drift. For all these reasons, the study of neuronal mechanisms affected by maternal care deprivation is also gradually paving the way to the search for therapeutic approaches aimed at interfering with the consolidation of maladaptive traits promoted by early life stress [103]. In this frame, animal models represent a fundamental tool to exploit this important research field.

Different stress paradigms of maternal deprivation are available, all of which revolve around the separation of the pups from the mother. These paradigms differ for the number of separation events, single separation of variable duration or multiple daily separations, or the critical developmental frame of maternal deprivation. Most frequently, separation is performed between postnatal days (PND) one and nine [104]. Generally, maternal deprivation triggers, in rodents, neophobic and anxiogenic effects, and quite often, it induces depressive traits. As shown below, maternal care deprivation leads to severe dysregulation of the HPA axis feedback, finally leading to a rapid increase in corticosteroid blood levels upon stress and an inherent increase of anxiety-like behaviors. In this regard, Schmidt and colleagues proved that each of the components of the HPA axis (from corticosterone, adrenocorticotropic hormone (ACTH), and CRH to Mineralocorticoid receptors (MRs) and GRs) responds to maternal deprivation at different time points with extremely specific time courses. These data suggest that during maternal deprivation, dynamic changes in all the components of the HPA axis happen [105]. Interestingly, maternal care deprivation also entails hypoglycemia, a dramatic decrease of leptin levels and hypothermia, further stressors potentially contributing to long-term effects on general and mental health [104].

### 7.2. Poor Parental Care

If parental care deprivation represents a form of stress that can trigger psychosis in adult life, poor quality parental care can similarly lead to an analogous pathological phenotype [103].

As described with maternal deprivation, the psychosocial stress that derives from poor parental care can also be modeled in rodents. Long-Evans rats dams differ in terms of the maternal care provided to pups. A subset of mothers indeed provides pups with poor care. The authors who selected them referred to these mothers as low licking and grooming mothers (Low-LG), while the others were defined as high licking and grooming (High-LG) (103). Poor maternal care severely impacts the neurodevelopment of pups in such a profound way that, once adult, they will show obvious behavioral abnormalities. Of particular note, (i) female pups of Low-LG mothers replicate similar behaviors with their own offspring acting in turn as Low-LG mothers, and (ii) these pups display excessive response to stress and limited stress-coping ability that can predispose them to psychiatric-like drifts. These behaviors do not seem to be of genetic origin. Indeed, as demonstrated when pups of Low-LG mother are raised by a High-LG mother, they can develop normally and provide good care to their own pups later on. 

Researchers have discovered that the inability to cope with stress displayed by Low-LG offsprings is the result of a molecular alteration in the hippocampus, an area of the brain that plays an important role in stress response and the processing of emotional and behavioral information [106]. Specifically, poor care leads to the transcriptional repression of the glucocorticoid receptor (GR), an important modulator of the HPA axis. The reduced expression of GR in the hippocampus is controlled by an epigenetic mechanism based on specific DNA methylation at the level of the GR promoter consensus sequence for the Immediate Early Gene EGR1. This leads to a reduced negative HPA axis feedback response to hematic corticosterone. As a result, these mice show heightened hormone blood levels and increased anxiety-like behavior. Remarkably, such an aberrant phenotype can be reverted using histone deacetylases (HDACs) inhibitors [103]. This stressful paradigm has not been used as widely as conventional maternal deprivation; nonetheless, it displays two fundamental advantages: (i) its construct validity and face validity are among the highest known in cellular and behavioral neuroscience, and (ii) this multifaceted model gives us the ability to shed new light on a wide range of processes induced by stress, extending from genes to behavior, thus providing a broader description of underlying molecular and epigenetic substrates of psychopathology [103]. 

### 7.3. Social Isolation

In human society, loneliness is a common condition and a strong predisposing factor for mood disorders. It has been shown that in old people, loneliness correlates with depressive disorders [107]. Similarly, social isolation negatively affects young subjects’ psychical equilibrium [108]. Different studies indicate, for instance, that auto-isolation, a common reaction of adolescents to certain stressful situations, including bullying, enhances psychopathological risk [109]. A pathological condition whose incidence has strongly increased in the internet era due to the rise of cyberbullying situations [110], is clinically linked to auto-isolation and has been recently proposed to be admitted into the DSM-5 [111,112]. Isolation-related stress supports the importance of social connections as functional to survival in mammals — the absence of a solid social network is a predisposing factor impacting emotional well-being [113]. As observed in humans also in rodent models, isolation itself represents a form of stress that may trigger psychiatric disorders featuring social refusal within a feed-forward process that ultimately increases the risk for the onset of psychopathologic conditions [114]. Because of the increasing incidence of psychiatric diseases associated with social isolation and refusal, animal models that recapitulate these conditions are needed to both improve our knowledge of underlying molecular mechanisms.

The behavioral effects of human social isolation can be simulated with the animal model of segregation [115]. In this paradigm of stress, adult mice, even if many studies also used adolescent (21 PND) or old (12–18 month) mice, are transferred from a cage where they used to live with other conspecific individuals, to an empty cage. This paradigm of stress is usually applied for variable time intervals that range from hours to a few weeks. When segregation is administered for a long time, mice show anxious and depressive phenotypes [114,115,116]. This pathological context, as in most cases, is further promoted by a chronic reiteration of the stressful source. Nonetheless, few-hour-long social isolation is enough to activate, although moderately, stress-response mechanisms such as the HPA axis and ANS [116]. What is more, it has been found that social isolation alters not only basal HPA axis activity, but also impairs glucocorticoid-mediated negative feedback after acute stress [117]. The activation of these systems supports physical and mental responses that are typical of the perception of a potential threat. From the evolutionary and ethological point of view, we speculate that hormonal response to isolation could be aimed at protecting the lone individual. Especially in mammals, social behavior holds many adaptive means; one of those, as an example, is related to decreasing vulnerability to attacks and predation. Interestingly, also because social isolation exerts profound HPA axis response modifications and affects behavior, chronic administration of social isolation is considered an animal model of several neuropsychiatric endophenotypes [117]. 

### 7.4. Social Defeat Stress

Bullying is becoming more and more common in our society [118]. Importantly, this stress affects both adolescents and adults being widespread in the army, common in workplaces (where it is known as mobbing) and within socially obliged communities such as neighborhoods. Another diffused form of bullying that sometimes does not emerge as it should is within families. It can happen that one of the two partners exerts a negative pressure against the other aimed at divesting him/her from decisional relevance, ultimately leading to the delegitimization of one of the spouses from decision making and everyday life management. The object of bullying faces repetitive and aggressive physical but also emotional abuses. Victims are usually identified as fragile and vulnerable individuals or, especially, when talking about mobbing, workers that are not free to react for many reasons, including psychological weakness in the context of power imbalance due to established hierarchical authority [119]. Once a bullying relation is structured, victims are systematically humiliated, and as a reaction, they tend to isolate themselves. Social isolation, together with the constant abuses, synergistically enhances the risk of psychiatric disorders such as depression. The devastating impact that this typology of stress has on individuals and its high incidence in modern societies strongly supports the need to investigate the neural mechanisms that are affected by bullying abuses. To this aim, animal models that mimic bullying are highly relevant in psychobiology research being continuously refined to better recapitulate inherent endophenotypes emerging in humans upon bullying experiences.

Social defeat stress (SDS) models bullying, being a chronic-stress paradigm that has been largely validated for the induction of depressive-like traits such as social avoidance or anhedonia in mice and rats [120,121]. From the molecular point of view, SDS induces prolonged activation of the HPA axis and alterations in 5-HT neurotransmission [122], processes vulnerable to overload that may play a role in the pathological abnormalities proper of depressive-like phenotype.

The peculiarity of this paradigm lies in the fact that it triggers pathological behavioral drifts only in a subset of animals that grossly corresponds to 40% of total tested animals, well-recapitulating human interindividual variability of behavioral responses [120]. These animals are indicated as susceptible or vulnerable. Conversely, those that do not show long-lasting pathological signs in response to the stress are called resilient [120,123,124]. Notably, the behavioral neutrality to stress, proper of resilient animals, does not only necessarily result from the lack of a negative response. Indeed, there are some pieces of evidence that indicate resilience as an active mechanism [123,125]. Thus, the study of biological bases of susceptibility, together with those of resilience, may help a better understanding of the pathogenic mechanisms at the basis of neuropsychiatric disorders. 

When SDS protocols are performed using inbred animals, they provide the chance to highlight the stochastic component of resilience and susceptibility. In addition, targeted genetic manipulation in inbred mice can help to unravel the possible contribution of specific genes and/or genetic elements in favoring or preventing stress vulnerability. 

Finally, animals that are susceptible to SDS represent an extraordinary model to study the effect of new antidepressant therapeutic strategies aiming to convert a vulnerable phenotype into a resilient one. All these features endow social defeat stress with remarkable scores in terms of construct and face validity [120], and at least for what concerns monoaminergic drugs, predictive validity [126].

We here describe the experimental details of a SDS protocol performed in mice [120], although, as said above, SDS can be effectively performed both in mice and rats with similar procedures [127].

The paradigm consists of 10 days of psychosocial stress perpetrated by an aggressor CD1 mouse on an experimental C57BL7/6 mouse. The size and weight of CD1 mice are 20% to 30% higher than C57BL/6 mice. In addition, CD1 mice are ex-breeding animals selected for an acquired aggressive profile. Each day of stress comprises two main phases during which target and aggressor mice interact through different modalities. In the first phase, direct physical interaction between the experimental C57BL/6 mouse and the aggressor CD1 mouse is allowed. During this phase, the aggressor mouse physically attacks the intruder. After five minutes, the two mice are separated and kept in visual and olfactory interaction. This represents the second phase of the SDS paradigm (indirect interaction), and it lasts the remaining 23 h and 55 min preceding the following physical interaction. Indirect interaction represents an important source of psychological stress for the experimental animal, still promoting neuronal mechanisms of the stress response. The social interaction with the aggressor CD1 mouse represents, for the experimental mouse, the stress of social defeat. At the end of the 10th day, experimental mice undergo a social interaction test [120] in which the tendency of the stressed mouse to interact with a naïve CD1 mouse, caged in a wire mesh cylinder, is scored. While control and resilient mice engage in social interaction, stress vulnerable mice do not. Also, the testing strategy displays good construct and face validity as social avoidance represents a core symptom of human depression. Notably, chronic stress that lasts less than 10 days is not capable of inducing long-term behavioral changes in rodents [120]. This evidence further supports the notion that the toxic consequences of bullying also depend on their reiterated administration. 

## 8. Ethical Considerations

As discussed in the previous paragraphs, animal models of stress can provide precious contributions to the investigation of neuropsychiatric disorders etiopathology and the discovery of new therapeutic strategies. Nonetheless, animal model employment has to be pursued within well-defined ethical frameworks, requiring accurate experimental planning not only from the scientific point of view but also considering crucial ethical issues [128,129].

In this respect, the guiding principles for the more ethical use of animals in scientific research are clearly illustrated by the ‘Three Rs’ (3Rs): Replacement, Reduction, and Refinement. Importantly, the three Rs for animal model usage in scientific research is indicated in the European directive 2010/63/UE on the “protection of animals used for scientific purposes”. The three R rules can be found at the following website: https://ec.europa.eu/environment/chemicals/lab_animals/3r/alternative_en.htm.

According to the principle of Replacement, researchers should consider alternative methods that avoid or replace the use of animals in their research. Although currently very far from being applicable within investigations on molecular, structural, and physiological brain correlates of behavior, alternative methods could be developed starting by in vitro models such as immortalized or primary cell cultures, including patients’ induced pluripotent stem cells (iPSC) and more complex systems such as organoids. However, once assumed that the animal model is strictly necessary, a frequent need in psychobiology, the Reduction principle should be applied. According to this principle, the smallest number of animals guaranteeing statistically significant data has to be employed as a result of a priori power analyses of group numerosity. In any case, researchers should always adopt methods to alleviate or minimize the potential pain, suffering, or distress that may derive from experimental procedures, with the final aim to maximize animal well-being. This last statement summarizes the principle of Refinement. For what concerns rodent stress paradigms, as at least in current years, it seems virtually impossible to replace in vivo strategies to gain information on molecular determinants of mammalian behavior. For this reason, many efforts must be undertaken to refine procedures. Indeed, if, on the one hand, the intrinsic severity of threatful paradigms poses serious ethical issues, on the other hand, researchers must do their best to render such procedures as tolerable as possible for the animals in order to avoid unwanted additional stressful conditions. To give some examples, animals administered with stress must be carefully observed by veterinary staff prior to and after the paradigms. When using swimming procedures, for instance, both water and room temperature has to be strictly controlled and kept warmer than usual. Animals must also be dried and controlled after stress administration. Similarly, thorough care must be provided to animals undergoing SDS within and after the procedures. Experimenters must avoid excessive aggressions to intruders and timely disinfect and treat any superficial wounds. In general, rodent health, which is anyway under constant control in animal facilities, has to be further guaranteed in the light of reaching animal well-being during all the moments that precede and follow the stressful procedures.

## 9. Conclusions and Future Perspectives

With the following words, the former director of the American National Institute of Mental Health (NIMH), Professor Stephen Hyman, gave us a concise overview of the current state of pharmacological research in the neuropsychiatric field: “Five decades of work on antidepressant drugs have not made them more likely to lift people out of depression (…), failures to improve efficacy reflect continued ignorance of the molecular mechanisms of depression (…) to find more-effective drugs that help more people, we must look for drugs that work in different ways” [13].

It is evident that in the next decades, researchers, aiming at identifying new, required therapeutic strategies, will move towards a deeper understanding of pathophysiological mechanisms that underlie the onset of depression as well as other neuropsychiatric disorders. New strategies will be suitable not only to simply interfere with symptoms, rather, targeting causative mechanisms, but they should also act as disease-modifying therapies.

We are aware of the limits of the animal model in the study of such complex pathologies. We cannot deny the extreme difficulty in comparing symptoms between different species (in this case, between humans and rodents), or the fact that animal models are, in the best-case scenario, just an approximation of the complexity of human behaviors. However, the animal model of stress can help us to both understand the molecular mechanisms underlying psychiatric disorders and to identify new pharmacological therapies. For example, from the rodent models, we have learned that acute stress can produce long-term behavioral effects only in case of extreme stressor severity, a condition that fosters neurobiological and behavioral endophenotypes of PTSD. Otherwise, acute stress is rapidly neutralized by adequate and physiological allostatic mechanisms [38,42,124]. Conversely, chronic stress seems to have a role in the etiology of neuropsychiatric behavioral alterations: This observation suggests that the homeostatic mechanisms that normally counteract acute stress can be, upon excessive load, corrupted, as they are vulnerable somehow [130,131,132]. This represents a fundamental discovery as, in this light, the systematic comparison of acute or chronic stress responses could lead to the discovery of new targets for pharmacological treatments. Moreover, much interesting information could be retrieved by analyzing the differential reactions to similar stresses administered at young, adolescent, or adult ages, not to mention gender differences in stress vulnerability [67]. 

As it often happens in scientific research, the major challenge is not the identification of a certain tool, but finding the best strategy to use it. We are thus confident that, if we ask the animal model the right question, administering them the most appropriate stress-paradigm to approach a specific problem, interpreting the molecular, circuitry, and behavioral responses in the frame of the RDoC-predicated common endophenotypes that can really be shared by rodent models and humans, such models integrated with neuroimaging studies based in humans, will make a difference in the future management of neuropsychiatric disorders.
